# Detection of *Mycobacterium avium* Subspecies *Paratuberculosis* from Intestinal and Nodal Tissue of Dogs and Cats

**DOI:** 10.1155/2013/323671

**Published:** 2013-09-23

**Authors:** Kate S. KuKanich, Javier Vinasco, H. Morgan Scott

**Affiliations:** Departments of Clinical Sciences (KuKanich) and Diagnostic Medicine/Pathobiology (Vinasco, Scott), Q-213 Mosier Hall, College of Veterinary Medicine, Kansas State University, Manhattan, KS 66506, USA

## Abstract

*Objective.* To determine prevalence of MAP in intestinal and nodal tissue from dogs and cats at necropsy at Kansas State University and to determine if an association existed between presence of MAP and gastrointestinal inflammation, clinical signs, or rural exposure. *Procedures.* Tissue samples were collected from the duodenum, ileum, and mesenteric and colic nodes of adult dogs (73) and cats (37) undergoing necropsy for various reasons. DNA was extracted and analyzed for insertion sequence 900 using nested PCR. Positive samples were confirmed with DNA sequencing. An online mapping system was used to determine if patients lived in an urban or rural environment based on the home address. Medical records were reviewed for clinical signs and histological findings at necropsy. *Results.* MAP was identified from 3/73 (4.1%) dogs and 3/37 (8.1%) cats. There was no documented association between presence of MAP and identification of histologic-confirmed gastrointestinal inflammation, gastrointestinal clinical signs, or exposure to a rural environment. *Conclusion and Clinical Relevance.* MAP-specific DNA can be identified within the intestinal and nodal tissue of dogs and cats that do not have pathological lesions or clinical signs consistent with gastrointestinal disease. The significance of this organism's presence without associated gastrointestinal pathology is unknown.

## 1. Introduction

*Mycobacterium avium* subspecies *paratuberculosis *(MAP) is the bacterial etiologic agent of Johne's disease, a severe chronic debilitating gastrointestinal disease of ruminants. Infection from this bacterium is responsible for substantial morbidity, mortality, and economic loss in cattle in the United States [1]. Within herds, MAP is transmitted mainly by ingestion of food or water contaminated with infected feces, but it can also be transmitted to offspring *in utero* or via infected milk [2, 3]. Clinical and histopathologic similarities between Johne's disease and Crohn's disease in humans, which is characterized by a chronic granulomatous ileocolitis, have led researchers to suspect MAP as an etiologic agent for Crohn's disease as well. Although MAP has been identified from clinical tissues of patients with Crohn's disease, Koch's postulates have not been proven to establish this link definitively [4, 5]. 

Investigation of MAP's potential contribution to gastrointestinal disease in dogs and cats is in its infancy. While both classic tuberculosis (including *M tuberculosis* and *M bovis*) and opportunistic mycobacteriosis (including *M fortuitum*) have been described extensively in dogs and cats, only one suspected clinical case of paratuberculosis has been reported in a dog [6]. The first study investigating MAP occurrence in the canine population detected MAP-specific DNA from intestinal biopsies of 8/42 (19%) dogs with gastrointestinal signs and 0/14 dogs without gastrointestinal signs, suggesting that MAP may be associated with gastrointestinal disease in dogs [7]. Further investigation into the prevalence of MAP in the general population of dogs and the relationship of MAP and gastrointestinal disease in dogs is warranted.

MAP research in cats is limited to 2 studies of nondomestic animals, including feral cats, from MAP-infected dairy farms [8, 9]. No information was reported regarding presence or absence of clinical disease in the cats from either study. In the Palmer study, 25 feral cats were trapped, euthanized, and necropsied, and MAP-specific DNA was identified by PCR from 7/25 cats' mesenteric nodes and 3/25 cats' ilea [8]. In the Corn study, 18 feral cats were trapped, euthanized, and necropsied, and MAP was isolated (via culture) from 2/18 cats' mesenteric nodes, 2/18 cats' ilea, and 1 cat's feces which suggests shedding [9]. The Corn and Palmer studies confirm that cats can be infected with MAP, but the prevalence of MAP in the general population of cats and its relationship with gastrointestinal disease warrant further investigation.

The objectives of this study were (1) to determine the prevalence of MAP in the duodenum, ileum, and mesenteric and colic lymph nodes of the general population of dogs and cats who underwent necropsy at our teaching hospital and (2) to determine if an association existed between detection of MAP and presence of histologically confirmed gastrointestinal inflammation, clinical signs, or exposure to a rural environment.

## 2. Materials and Methods

### 2.1. Study Population

This was a cross-sectional study to determine the prevalence of MAP in intestinal and nodal tissue from dogs and cats submitted for necropsy at the Kansas State Veterinary Diagnostic Laboratory between September 2009 and April 2011. Animals were required to be at least two years of age.

### 2.2. Tissue Collection

At necropsy, up to a 2-inch section of proximal duodenum, distal ileum, mesenteric lymph node, and colic lymph node was aseptically collected and individually stored and frozen. Tissue samples were thawed prior to processing, and 40 mg of each tissue type was aseptically obtained for DNA isolation. 

### 2.3. DNA Extraction

 DNA extraction was performed by a modified version of a previously described method using the DNeasy Blood and Tissue kit [7] (Qiagen, Inc. Valencia, CA). In brief, 40 mg of tissue was mixed with 180 mL of lysis buffer and incubated at 37°C overnight. Fifty microliters of Proteinase K and 200 *μ*L of the second lysis buffer were added to the suspension, and the suspension was incubated overnight at 70°C with continuous shaking. The DNA extraction was continued in the automated system Qiacube (Qiagen, Inc. Valencia, CA). The final elution was 100 microliters. For quality assurance, DNA was analyzed by spectrophotometry (Nanodrop ND-1000, NanoDrop Technologies Inc. Wilmington, DE). 

### 2.4. Nested PCR

 Nested PCR was performed on isolated DNA using primers based on the insertion sequence IS900 (Table 1) [10]. The first round of PCR amplified a fragment of 572 bp; the second round amplified a fragment of 452 bp. A Master Mix Kit (HotStarTaq Master Mix kit, Qiagen, Inc. Valencia, CA) was used in both amplifications. Both PCRs were set up using the Qiagility robotic system (Qiagility, Qiagen, Inc. Valencia, CA). PCR products were evaluated by microcapillary electrophoresis using Qiaxcel (Qiaxcel, Qiagen, Inc. Valencia, CA). To minimize risk of contamination of PCR samples, routine precautions were employed, including having a HEPA filter present in the instrument, UV radiation, and cleaning with DNaway after each procedure. The positive control was a DNA sample from a MAP isolate cultured from a clinical sample, and the negative control was Qiagen DNase free water. Control samples were not located near test samples on the testing plate.

### 2.5. DNA Sequencing

 PCR products that were positive for MAP by nested PCR were purified using Wizard SV gel and PCR Clean-Up System (Promega, Madison, WI). DNAs were sequenced using an Applied Biosystems 3730 DNA Analyzer. Both the sense and antisense strands of each PCR product were sequenced. The electropherograms were analyzed using Chromas Lite 2.1 software, and the DNA sequences were subjected to BLAST analysis of GenBank.

### 2.6. Medical Record Review

Necropsy reports were reviewed when available to determine if patients had histopathological evidence of inflammatory gastrointestinal disease. Medical records were reviewed to determine if vomiting, diarrhea, decreased appetite, and/or weight loss were recorded as part of the patient's clinical signs or history at the time of death. An online mapping system with satellite viewing (Google Maps) was used to determine whether patients lived in an urban or rural environment, based on their home address. An environment was labeled as urban if the home was in a neighborhood or other highly populated or commercial area. An environment was labeled as rural if the home was in the country or on a farm. 

### 2.7. Statistical Analysis

Fishers exact tests were performed to compare proportions of animals whose tissue tested positive for MAP with presence of inflammatory gastrointestinal disease, with presence of gastrointestinal clinical signs, and with exposure to rural environment. A commercial statistical software program was used for all comparisons (SigmaPlot 12.0, Systat Software, Inc. San Jose, CA). A *P* value ≤ 0.05 was considered significant. 

## 3. Results

Seventy-three dogs and thirty-seven cats over two years of age were presented for necropsy between September 2009 and April 2011 and were included in this study. The median age of dogs was 8 years old (range 2–15 yrs), and there were 63 purebred dogs and 10 mixed-breed dogs. The median age of cats was 14 years, and there were 7 purebred cats (4 Siamese, 2 ragdolls, and one Abyssinian). The remaining cats were domestic short-haired and long-haired cats. 

Three of seventy-three dogs (4.1%) were positive for MAP by PCR, confirmed by DNA sequencing (Table 2). After analysis in the NCBI-BLAST database of GenBank, the DNA sequences were nearly identical (99.0%) to IS900. MAP was identified from the distal ileum in two dogs and the mesenteric lymph node in one dog; no dog had multiple tissues positive for MAP. Of the 48 dogs with histopathology being performed on the gastrointestinal tract at necropsy, 10 dogs had gastrointestinal inflammation, including ulcerative disease (3 dogs), lymphoplasmacytic enteritis (2 dogs), infection (2 dogs), necrosis (2 dogs), and neoplasia (1 dog). No dog with inflammatory gastrointestinal disease had a tissue positive for the presence of MAP, and no association was identified between histopathologic inflammation and presence of MAP in dogs (*P* = 1.000). In the dogs from which MAP was identified, 0/3 had vomiting, diarrhea, decreased appetite, or weight loss documented in the medical record; in the remaining enrolled dogs, vomiting was recorded in 15/70 dogs, diarrhea in 7/70 dogs, decreased appetite in 24/70, and weight loss in 10/70. No significant associations were identified between presence of any of these 4 clinical signs and presence or absence of MAP in dogs (*P* > 0.05 for each comparison). Seventeen dogs lived in a rural environment and 55 in an urban environment, and 1 came from a humane society and was thus not classified as rural or urban. In dogs, no association was found between identification of MAP in intestinal or nodal tissue and exposure to a rural environment (*P* = 0.560). 

Three of thirty-seven cats (8.1%) had nodal tissue positive for MAP by nested PCR, confirmed by DNA sequencing (Table 2); as with the canine samples, after analysis in the NCBI-BLAST database of GenBank, the DNA sequences from feline samples were nearly identical (99.0%) to IS900. MAP was identified from the colic node in two cats and the mesenteric node in one cat. No cat had MAP identified from intestinal tissue or multiple tissues positive for MAP. Of the 23 cats with histopathology of the gastrointestinal tract, eleven had evidence of gastrointestinal inflammation, described as neoplastic (4), lymphoplasmacytic (3), eosinophilic (2), pyogranulomatous (1), and ulcerative (1). One cat with inflammatory gastrointestinal disease (carcinoma) had colic lymph node test positive for presence of MAP, but no statistical association was present between histopathologic gastrointestinal disease and MAP in cats (*P* = 1.000). Of the cats from which MAP was identified, 1 cat was reported to have vomiting, decreased appetite, and weight loss, 1 cat had decreased appetite, and the third cat was reported to have none of these signs. In comparison, of the remaining cats in the study, 5/34 had vomiting, 2/34 had diarrhea, 13/34 had decreased appetite, and 8/34 had weight loss documented in the medical record at the time of death. No significant associations were present between presence of any of these 4 clinical signs and presence or absence of MAP in cats (*P* > 0.05 for each comparison). Nine cats lived in a rural environment, twenty-seven cats lived in an urban environment, and home address was unavailable for one cat. Information regarding whether cats spent time indoors or outdoors was unavailable. In cats, no association was found between identification of MAP from nodal tissue and exposure to a rural environment (*P* = 0.148).

Considering the differences in prevalence between dogs (4.1%) and cats (8.1%), these did not differ in a meaningful or significant (*χ* ^2^_(1)_; *P* = 0.370) way.

## 4. Discussion

This study confirmed that the prevalence of MAP-specific DNA in the overall population of dogs (4.1%) and cats (8.1%) presenting for necropsy in Kansas is low. This prevalence is similar to that reported for local beef cattle (5 ± 2%) and dairy cattle (8 ± 3%) [11]. 

Previously, MAP-specific DNA has been identified from the gastrointestinal tissue of 8/42 dogs with chronic gastrointestinal disease, but MAP-specific DNA was not identified from any dog who did not have gastrointestinal disease, suggesting that MAP may be associated with gastrointestinal disease in dogs [7]. In contrast to the Glanemann study, the present study documents the identification of MAP from dogs with no evidence of clinical or histopathologic gastrointestinal disease.

Previous studies have isolated MAP and identified MAP-specific DNA from feral and farm cats; in these studies histological lesions consistent with paratuberculosis were absent and clinical signs were not reported [8, 9]. In the present study, MAP was identified from one cat with a chronic inflammatory GI disease (carcinoma) that showed clinical signs of vomiting, decreased appetite, and weight loss. The remaining two cats with MAP did not have histological changes of their gastrointestinal tract. No association was made between presence of clinical signs and identification of MAP-specific DNA in cats in this study.

The significance of finding molecular evidence of MAP in the intestinal and nodal tissue of dogs and cats without concurrent evidence of gastrointestinal pathology is difficult to interpret. Possible explanations are that this organism may be nonpathogenic in some animals or potentially have a prolonged incubation period. Although this study found MAP to be uncommon in this population of animals, further investigation into the possibility of either intermittent or persistent fecal shedding of MAP by canine and feline carriers may be warranted to better understand a potential public health risk. 

There were numerous limitations in this study. The sampled population of dogs and cats having necropsies may have selected for unique diseases and does not necessarily reflect the general population of dogs in Kansas or the rest of the country. Not all the patients that were necropsied had histopathology performed, and special staining for acid fast bacteria (Ziehl Nielsen) was rarely reported and not performed on any dog or cat with tissue positive for MAP. Despite the sample size, the number of dogs and cats with gastrointestinal inflammation was unfortunately small, as was the number of patients coming from a rural environment. Determining whether a pet lived in a rural versus urban environment based on home address does not necessarily equate to exposure to farm animals, because it does not account for animals who have ability to roam nor provide information on whether cats spend time indoors or outdoors. 

## 5. Conclusions

MAP-specific DNA was identified from the intestinal and nodal tissue of 3/73 (4.1%) dogs and 3/37 (8.1%) cats; the prevalence did not differ significantly between these two host species (*χ* ^2^_(1)_; *P* = 0.370). There was no documented association between presence of MAP-specific DNA and presence of gastrointestinal inflammation, clinical signs, or exposure to a rural environment. MAP-specific DNA can be identified in the intestinal and nodal tissue of dogs and cats that do not have clinical signs or gastrointestinal pathology. The significance of this organism's presence without associated abnormal gastrointestinal pathology is unknown. 

## Figures and Tables

**Table 1:** Primers used for nested PCR (modified from Vansnick et al., 2004 [10]).

Primer	DNA sequence	Product size
IS900S1	ggg ttg atc tgg aca atg acg gtt a	572 bp
IS900R3	agc gcg gca cgg ctc ttg tt	

IS900S2	gga ggt ggt tgt ggc aca acc tgt	452 bp
IS900R1	cga tca gcc acc aga tcg gaa	

**Table 2:** Species, signalment (age, sex/neuter status, breed), histopathologic disease, home environment, and tissue from which MAP-specific DNA was isolated.

Species	Signalment	Histopathology	Home environment	Tissue from which MAP DNA was identified
Feline	19 yr FS DSH	Interstitial nephritis; pancreatitis	Urban	Colic node
Feline	16 yr FS DLH	Membranous glomerulonephropathy; biliary cystadenoma	Rural	Mesenteric node
Feline	20 yr MN DSH	Intestinal carcinoma	Rural	Colic node
Canine	5 yr FS Mastiff	Malignant histiocytosis of the spleen	Urban	Distal ileum
Canine	5 yr FS Pug	Renal hypoplasia; glomerulonephritis	Urban	Mesenteric Node
Canine	7 yr MN Labrador	Pneumonia	Rural	Distal ileum

yr: year; FS: female spayed; DSH: domestic short haired cat; DLH: domestic long haired cat; MN: male castrated; GI: gastrointestinal; MAP: *Mycobacterium avium *subspecies* paratuberculosis*.

## Abbreviations

MAP: *Mycobacterium avium* subspecies *paratuberculosis*
PCR: Polymerase chain reaction.
